# Domain-wall pinning and defect ordering in BiFeO_3_ probed on the atomic and nanoscale

**DOI:** 10.1038/s41467-020-15595-0

**Published:** 2020-04-09

**Authors:** Andreja Bencan, Goran Drazic, Hana Ursic, Maja Makarovic, Matej Komelj, Tadej Rojac

**Affiliations:** 1 Electronic Ceramics Department, Jozef Stefan Institute, 1000 Ljubljana, Slovenia; 2 Jozef Stefan International Postgraduate School, 1000 Ljubljana, Slovenia; 3 Department of Materials Chemistry, National Institute of Chemistry, 1000 Ljubljana, Slovenia; 4 Department for Nanostructured Materials, Jozef Stefan Institute, 1000 Ljubljana, Slovenia

**Keywords:** Materials science, Condensed-matter physics, Ferroelectrics and multiferroics

## Abstract

Electro-mechanical interactions between charged point defects and domain walls play a key role in the functional properties of bulk and thin-film ferroelectrics. While for perovskites the macroscopic implications of the ordering degree of defects on domain-wall pinning have been reported, atomistic details of these mechanisms remain unclear. Here, based on atomic and nanoscale analyses, we propose a pinning mechanism associated with conductive domain walls in BiFeO_3_, whose origin lies in the dynamic coupling of the p-type defects gathered in the domain-wall regions with domain-wall displacements under applied electric field. Moreover, we confirm that the degree of defect ordering at the walls, which affect the domain-wall conductivity, can be tuned by the cooling rate used during the annealing, allowing us to determine how this ordering affects the atomic structure of the walls. The results are useful in the design of the domain-wall architecture and dynamics for emerging nanoelectronic and bulk applications.

## Abstract (web summary)

Defects play an important role in the pinning of domain walls (DW) in ferroelectric materials but the underlying mechanism is still elusive. Here, the authors report on the dynamic interaction between electronic defects and DWs in BiFeO_3_ resulting not only in pinning but also affecting the DW conductivity.

## Introduction

The unique functionalities of topological defects in ferroic materials offer unlimited possibilities for nanoscale applications^1^. In particular, electrically conductive ferroelectric and ferroelastic domain walls (DWs) have recently been identified as functional entities that could enable a new era of nanoelectronic devices^2^. The reason is that such interfaces can be displaced, written and erased by external electrical^3^ or stress fields^4^. Coupled with their electrical conductivity, which can be engineered^5–7^, spatially tunable DWs could provide a controllable way to design conductive paths on the nanoscale^8^, resulting in DW diodes^9^, memories^10,11^ and switches^12^. These same conductive features when present in polycrystalline bulk ferroelectrics, as recently demonstrated for BiFeO_3_, can generate a large electromechanical nonlinearity and hysteresis^13^, which are crucial for actuators, sensors and ultrasound devices relying on bulk piezoelectrics^14–16^. While impressive progress has been made in the nanotechnology, engineering and macroscopic implications of conductive DWs, little has been done to understand the atomistic details of their dynamics under applied electric fields. A better insight into these mechanisms is therefore necessary to find the fundamental limits of future nanoscale and bulk devices, either relying on or operating with conductive DWs.

Since the discovery of conductive DWs in multiferroic BiFeO_3_ thin films, it has been suggested that the local conductivity should be enhanced by the presence of charged point defects accumulated inside DW regions^3^. Different type of defects, including oxygen vacancies, bismuth vacancies, electrons and electron holes, have been discussed for BiFeO_3_^17–21^, naturally indicating the key role of the defect chemistry of the ferrite and thus the processing conditions (such as the temperature and partial pressure of oxygen)^21,22^. In the case of air-processed polycrystalline BiFeO_3_, the accumulated defects at the head-to-tail DWs were directly identified by atomic-resolution microscopy as Bi vacancies and electron holes^21^. The latter were revealed to be localized at Fe^3+^ sites, creating Fe^4+^ oxidized states, and were experimentally confirmed to be responsible for the p-type DW conduction in polycrystalline BiFeO_3_. To be noted is that macroscopic p-type conduction has also been confirmed in chemically derived BiFeO_3_ thin films and is thus not only confined to polycrystalline BiFeO_3_^23^. A reasonable assumption is that the accumulated defects will result in DW pinning effects, affecting the DW dynamics, such as short-range displacements under applied electric field. Owing to the particular p-type nature of the defects and their location, however, this pinning mechanism has not yet been investigated. In addition, the mechanism is expected to be different from that in prototypic ferroelectrics, such as acceptor-doped hard Pb(Zr,Ti)O_3_ (PZT) and BaTiO_3_, in which the strong DW pinning is controlled by acceptor–oxygen-vacancy defect complexes^24–27^.

In addition to the type of defects, the DW pinning strongly depends on the spatial distribution of defects, often conceptualized in terms of the “order/disorder” defect state^16,28,29^. While lacking a proof at the atomic scale, macroscopic data on the polarization switching in hard PZT-^16,29–31^ and BiFeO_3_-based^32–35^ ferroelectrics suggest that the ordering degree of the defects can be controlled by the cooling rate used during the annealing. This means that quenching of the sample from above the Curie temperature (*T*_c_) induces defect disorder, while slow cooling favours defect ordering. Just like the electric-field cycling^36^, quenching is thus a valuable method to control defect distribution in hard ferroelectrics. Considering that in BiFeO_3_ the defects, at least in part, tend to accumulate at the DWs (a process that in this study is subsequently referred to as “defect ordering”, similar to ref. ^29^), applying the concept of quenching to controllably accumulate and deplete the defects to/from DWs provides a unique opportunity to explore the implication of these defects in the atomic structure and electrical conductivity of DWs.

In this study we adopt a previously reported methodology^21^ based on atomic-scale scanning-transmission electron microscopy (STEM) coupled with piezo-response and conductive atomic-force microscopy (PFM, c-AFM) to probe the interactions between the accumulated defects and the DWs in polycrystalline BiFeO_3_. The results show that under applied electric field the mobile electron holes can follow domain-wall displacements, suggesting that the displacements at subswitching fields are most probably controlled by p-type polaron hopping. Next, we confirm on the atomic scale that the quenching leads to the depletion of the defects from the DWs and, inversely, that these defects tend to reaccumulate at the DWs by slow cooling (aging). This time- and temperature-dependent process increases the fraction of conductive DWs and might be thus an important factor for the reliability and stability of the DW conductive paths in, e.g., future DW-based devices. Finally, the comparison of the DW structure with the accumulated (pristine state) and the depleted defects (quenched state) directly reveals the strong influence of the defects and the associated chemical strain on the DW thickness and the local strain fields within and near the DW regions.

## Results

### Redistribution of defects at DWs by electric field

**Fig. 1:**
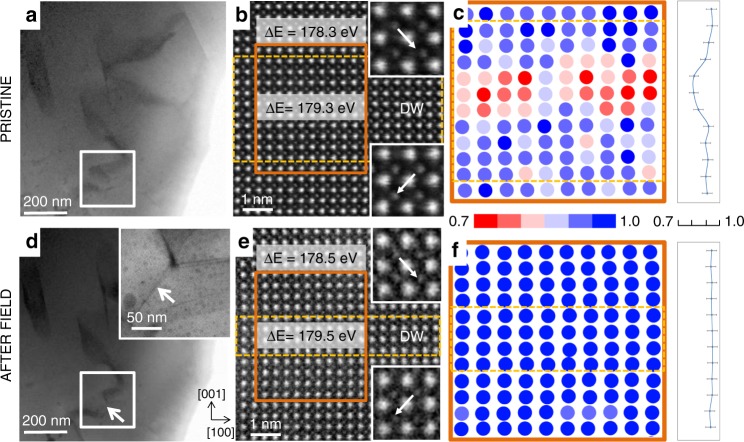
Redistribution of defects at DWs by application of electric field as observed on the atomic scale. Images of domains and atomic-scale DW analysis **a**–**c** before and **d**–**f** after the application of the electric field to the BiFeO_3_ sample. Bright-field (BF) STEM images of domains in the same region in BiFeO_3_ before and after the application of the electric field are shown in panels **a** and **d**, respectively. The sample region where significant changes were observed after the field application is highlighted with white boxes. The inset of panel **d** shows an enlarged view of the newly formed DW (marked with arrow), appearing as a result of the applied electric field. HAADF-STEM image in [010] zone axis of a 109° DW before the application of the electric field and of the newly formed 109° DW after field application is provided in panels **b** and **e**, respectively. The insets of these panels show the Fe displacement directions (relative to the Bi sublattice) in the two adjacent domains where dashed-yellow boxes indicate DW regions. Experimental EELS spectra were acquired on and off these DW regions, as indicated by grey bands in panels **b**, **e** with labelled energy onset difference (ΔE) between the O-K and Fe-L_3_ edges. Normalized distribution maps of Bi-column intensities with the corresponding average intensity line profiles before and after the application of the electric field are shown in panels **c** and **f**, respectively. These regions correspond to those marked with full orange boxes in panels **b, e**. Individual colours in the scale bar represent 0.05 steps in Bi-column intensities. Error bars in the intensity line profiles in panels **c**, **f** represent the standard deviation of the intensity measurements performed inside domain regions (~4% relative).

We first present the results of an ex situ electric-field STEM experiment in which a BiFeO_3_ sample prepared for STEM analysis (Supplementary Fig. 1) was analysed prior to and after the application of an electric field. The analysis of a head-to-tail 109° DW in the sample before field application (referred to as the pristine state) is presented in Fig. 1a–c. The DW region was identified by extracting the relative off-centre Fe displacements for each projected unit cell across the sample region (insets and arrows in Fig. 1b). The point defects were analysed by quantitative high-angle annular dark-field (HAADF) and electron energy-loss spectroscopy (EELS) analyses. While all the details of these analyses are reported in ref. ^21^, we briefly re-explain them for clarity.

To identify the oxidation state of Fe, we adopt EELS using the energy onset difference (ΔE) between the O-K and Fe-L_3_ edges^37^. The different ΔE values measured inside (179.3 eV) and outside (178.3 eV) the DW region in Fig. 1b suggest the presence of Fe^4+^ oxidation states inside the DWs with the surrounding domains predominantly consisting of Fe^3+^. Statistical ΔE data determined on different BiFeO_3_ samples and ΔE reference data determined on standard materials are reported in Supplementary Fig. 2. We also quantified the Bi-column intensities from HAADF images^38^ and extracted the normalized Bi-column intensity map shown in Fig. 1c. The significant reduction in the Bi intensities inside the DW region (~20%) arises due to missing Bi atoms and thus reveals the presence of Bi vacancies (reddish circles in Fig. 1c and the drop of Bi intensity at the DW seen in the adjacent profile).

Defect accumulation at the DWs in polycrystalline BiFeO_3_ was previously found at all three head-to-tail rhombohedral DW variants (71°, 109° and 180°)^21^. A number of experimental and theoretical studies support the identified Fe^4+^ and Bi vacancies, i.e. (i) the p-type conduction of air-sintered BiFeO_3_ associated with Fe^4+^ states (i.e. electron holes localized at Fe^3+^ host sites), as implied by the defect-chemistry model^39–41^, (ii) density functional theory (DFT) calculations, predicting a low formation energy of Bi vacancies and the corresponding p-type conduction in oxidizing conditions^42–44^, (iii) synchrotron X-ray absorption spectroscopy (XAS) studies where a shift to higher energies of the Fe K-edge in Co-doped BiFeO_3_, relative to the Fe K-edge position in Fe_2_O_3_, confirmed the presence of Fe^4+^ states in the BiFeO_3_ samples^45^.

We now show how the identified defects redistribute with the applied electric field. First, we imaged the same sample region before (Fig. 1a) and after (Fig. 1d) the ex situ application of the electric field. As observed in Fig. 1d (arrow, inset), we identify a new 109° head-to-tail DW that was not observed before the field was applied (compare white box in Fig. 1a, d). The EELS analysis on this newly created DW resulted in nearly the same ΔE values as those measured at the 109° DW in the pristine sample (compare the values in Fig. 1b, e), suggesting the presence of Fe^4+^ states (electron holes) inside the switched DW. In contrast, no evidence of Bi-vacancy accumulation was found, as seen by the homogeneous Bi-column intensities and flat Bi intensity profile (Fig. 1f). The results reveal that, unlike Bi vacancies, electron holes tend to follow the DW switching by reaccumulating inside the wall. This observation can be rationalized in terms of the room-temperature electrical mobility of Bi vacancies, which can safely be assumed to be orders of magnitude lower than that of the electron holes^21^. Therefore, while the mobile electron holes dynamically couple to the switched DW, the rather immobile Bi vacancies cannot do so, at least not to the same extent; at most, they migrate by hopping over short distances, possibly between neighboring lattice sites (further support is provided in Supplementary Fig. 3).

The reaccumulation of electron holes (Fe^4+^) implies that the switched DWs should still show the p-type conductive character. This is confirmed by the previous identification of conductive DWs in poled BiFeO_3_ samples^13^ and by the identification of a conductive DW that was lithographed in the sample analysed in this study (Supplementary Fig. 4). Similarly, electrostatic force microscopy (EFM) imaging confirms the same electrostatic configuration at the DWs before and after switching with an electric field (Supplementary Fig. 5).

### Redistribution of defects at DWs by quenching and aging

After examining the redistribution of the charged point defects by the electric field, here we investigate a similar redistribution, this time induced by quenching. The concept of the method can be divided into two stages^16,28–30^. Initially, the sample consisting of defects in ordered states (in our case represented by the defects accumulated at the DWs; see Fig. 1a–c) is annealed at temperatures above *T*_c_. Due to the absence of spontaneous polarization in the paraelectric phase and driven by the thermal energy, the defects tend to rearrange ideally into a disordered state by diffusion, depleting from the locations of the original DWs. In the second stage, to freeze this disordered defect state and prevent reordering of the defects by diffusion during slow cooling, the sample is quickly cooled (quenched) to room temperature. The common manifestation of this order-disorder defect transition is de-pinching and de-biasing of the original polarization–electric-field (*P*–*E*) hysteresis loops upon quenching, as previously shown for hard PZT^28–30^^,^ and BiFeO_3_^32^ (such changes are also observed for the BiFeO_3_ samples analysed here; see Supplementary Fig. 6).

**Fig. 2:**
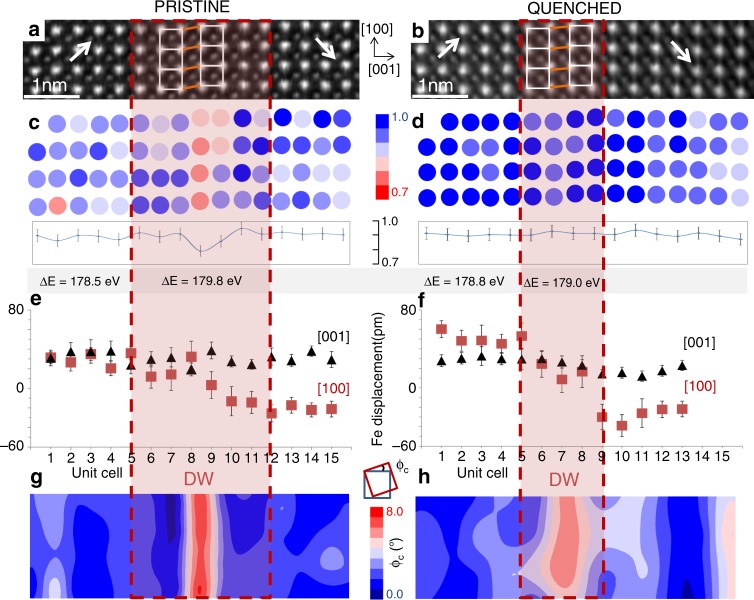
Defect analysis and atomic structure of DWs in pristine and quenched BiFeO_3_. HAADF-STEM image in [010] zone axis of a 109° DW in **a** pristine and **b** quenched BiFeO_3_ with corresponding **c**, **d** normalized distribution map of Bi-column intensities and average intensity profiles across the DW region; the values of ΔE between the O-K and Fe-L_3_ edges measured on and off the DW are also shown in the panels **c**, **d**; **e**, **f** average of Fe displacements determined from individual unit-cell rows and measured along [001] and [100] directions; **g**, **h** lattice-strain map represented in terms of lattice-distortion angle measured in [001] direction (*ϕ*_c_). The DW region is indicated by a dashed red box. In panels (a,b) the arrows indicate the direction of the Fe displacements from the centre of the Bi sublattice; enlarged distances between the sheared Bi atoms columns (shear lattice strain) inside the DWs are indicated by red lines. Error bars in the intensity line profiles in panels **c**, **d** represent the standard deviation of the intensity measurements performed inside domain regions (~4% relative). Error bars in panels **e**, **f** represent the standard deviation of the displacement measurements across the DW.

Figure 2 compares the structure and chemistry of a DW in the pristine (left figure column) and quenched state (right figure column). We specify that the analysis was done on two different DWs in the pristine and quenched sample, though both are 109° DWs. The reason is that the samples are quenched from above *T*_c_, meaning that the domain structure will likely rearrange into a new configuration. The domain structure formation in quenched samples should also be affected by the disordered defect state set in the paraelectric phase. Therefore, it is expected that the DWs after quenching to room temperature will attain positions inside the grains different from those in the original, pristine sample. While this prevent us to analyze the exactly same DW before and after the quenching process, the results, which are shown next, still confirm the quenching-induced defect rearrangement at DWs on the atomic scale.

Consistent with the macroscopic *P*–*E* measurements (Supplementary Fig. 6), quenching indeed provokes the redistribution of defects at the DWs. This is clearly seen by comparing the Bi-column intensity maps of the two DWs (marked with a dashed red box in Fig. 2): while the pristine DW contains Bi vacancies (reddish circles in the middle of the DW in Fig. 2c and the corresponding drop of the Bi intensity seen in the profile), no such accumulation is observed after quenching (blue circles in Fig. 2d with no drop of the Bi intensity at the DW in the profile). Similarly, the EELS analysis of the quenched sample reveals a much smaller difference in the ΔE values measured inside and outside the DW region (0.2 eV difference; Fig. 2d) as compared to that in the pristine sample (1.3 eV difference; Fig. 2c), indicating a reduced concentration of Fe^4+^ at the DW after quenching (further details regarding Fe^4+^ defects are reported in Supplementary Fig. 2). As it will be analyzed later, this depletion of defects from the DWs have a profound effect on the internal atomic structure of the walls (see next section, which further discusses the rest of the results shown in Fig. 2e–h).

After confirming the depletion of the defects from the DWs induced by quenching, we continue our analysis by investigating the effect of this depletion on the local DW conductivity. Here, we consider two observations from the literature. The first is that the DW conductivity in air-sintered polycrystalline BiFeO_3_ is p-type and thus related to polaron hopping of the accumulated mobile electron holes^21^, identified as oxidized Fe^4+^ states (see Fig. 2, pristine sample). We note that such a scenario, in which accumulated mobile defects dominate the local conductivity, has also been discussed for thin-film BiFeO_3_^3,17–19,22^ and PZT^46^. The second observation, evidenced by macroscopic measurements of the polarization switching in BiFeO_3_^32^, implies a link between the cooling rate (used during the sample annealing) and the degree of defect ordering. As the fast cooling (quenching) prevents defects from ordering, resulting in DWs depleted of defects (as confirmed in Fig. 2d), slower cooling rates should provide sufficient time for the migration of defects and their redistribution in equilibrium ordered states. For the BiFeO_3_ samples analysed here, this defect reordering from the quenched state was indirectly confirmed by the reappearance of pinched and biased *P*–*E* loops after the quenched BiFeO_3_ sample was slowly cooled (0.5 °C/min) from 760 °C (a process herein referred to as the aging;^16,28,32^ Supplementary Fig. 6). Therefore, considering these two aspects we postulate that the DW conductivity in BiFeO_3_, being dominated by the ordering degree (accumulation) of the electron holes at the walls, will be affected by the rate at which the samples are cooled from the annealing temperature.

**Fig. 3:**
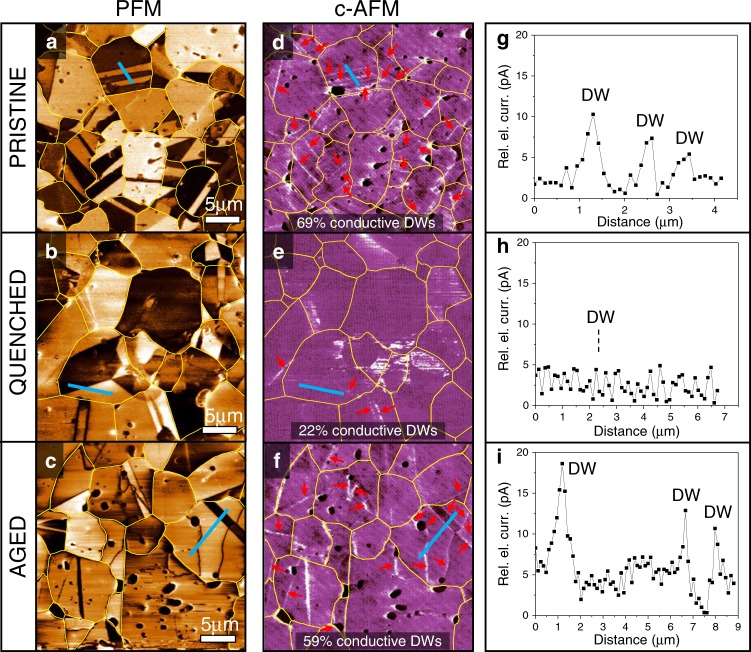
Electrical-current signals at DWs in pristine, quenched and aged BiFeO_3_. **a**–**c** PFM out-of-plane amplitude images, **d**–**f** c-AFM maps and **g**–**i** electric-current profiles corresponding to **a**, **d**, **g** pristine, **b**, **e**, **h** quenched and **c**, **f**, **i** aged sample. The red arrows in the c-AFM images indicate the DWs with enhanced electric-current signal. The fraction of conductive DWs in the three samples is indicated on the respective c-AFM maps (this fraction was determined statistically on several sample regions by probing up to ~200 DWs per sample). The blue lines in the PFM and c-AFM images correspond to the distance along which the electric-current profiles, shown in panels **g**–**i**, were extracted. The DW positions along this distance are indicated on the profiles (DW—domain wall). The data are shown in units of relative electrical current, normalized to the lowest background signal arising from the domains. The grains in the PFM and c-AFM images were outlined to make the domains and DWs easier to visualize.

To tackle the problem, we use c-AFM mapping, analysing the pristine, quenched and aged samples. The aged sample refers to the quenched BiFeO_3_ that was additionally cooled with the slow rate (0.5 °C/min). PFM images and the corresponding c-AFM maps of regions with domains in these three samples are shown in Fig. 3a–c and Fig. 3d–f, respectively. As can be clearly observed in the c-AFM map in Fig. 3d (see red arrows), the pristine sample shows an enhanced electrical-current signal (Fig. 3g) pertaining to a large fraction of the analysed DWs (the domains and DWs can be identified by the PFM contrast in Fig. 3a). It is worth noting that not all of the DWs show an obvious current signal exceeding the background level, which is expected considering the likely variation in the local conductivity from wall to wall (probably depending on the local defect concentration and the statistical distribution of defects inside the domains and at the walls, like shown for the defect-related DW thickness distribution in PbTiO_3_ single crystals^47^).

In the quenched sample, a much smaller fraction of the DWs exhibit a conductive character (see red arrows in Fig. 3e and, as an example, the current profile in Fig. 3h showing no obvious peak at the DW position). This observation is consistent with the observed depletion of Fe^4+^ states (mobile holes) from the DWs (see similar ΔE values determined on and off the DW in Fig. 2d and Supplementary Fig. 2 for supporting data). Note that the larger bright areas in Fig. 3e inside the domains most likely arise from the widely discussed AFM-tip-induced Schottky-barrier effects, which depend on the orientation of the polarization^13,48,49^. Another possible explanation is the uneven distribution of charges, resulting in conductive regions over larger domains areas. Nevertheless, these current signals are clearly not concentrated at the DWs.

Finally, aging of the quenched sample restores a larger fraction of conductive DWs (red arrows in Fig. 3f), resulting in an average conductive state similar to that in the pristine sample. This suggests a dynamic reordering of the mobile electron holes at the DWs during aging, which is consistent with the statistically identified Fe^4+^ states at the walls in the aged sample using EELS (Supplementary Fig. 2). We also note that this electron-hole reaccumulation is coupled to the concurrent reaccumulation of Bi vacancies (Supplementary Fig. 7). Therefore, aging of the quenched sample tends to restore the initial state with the DW-accumulated electronic and ionic defects.

Considering the large number of DWs present in typical millimetre-sized bulk polycrystals, we endeavoured to obtain a statistically more relevant picture of the conductive state of the DWs in our bulk BiFeO_3_ samples. This was done by analysing several c-AFM maps in different sample regions using a range of tip-bias voltages (see Methods) and relying on the electric-current signals determined at DWs (examples of such signals are shown in the current profiles in Fig. 3g–i). After probing a total of ~200 DWs in each of the three samples, the respective fractions of DWs exhibiting current signals were extracted to 69% (pristine), 22% (quenched) and 59% (aged) (also indicated in Fig. 3d–f). Note that the quenched sample still contains a measurable fraction of conductive DWs, which could be attributed to the imperfect disordering induced by the quenching and/or partial electron-hole reaccumulation at the DWs occurring to a smaller degree at room temperature after the sample was quenched. From a relative perspective, however, the decreased fraction in the quenched sample, relative to the pristine state, and its increase upon aging, satisfactorily agrees with the expected electron-hole ordering degree.

### Influence of defect accumulation on the DW atomic structure

Motivated by the unique comparison of the same type of DW (109°) with systematically accumulated and depleted defects in the pristine (Fig. 2c) and quenched sample (Fig. 2d), respectively, we analyse here how this different defect distribution affects the DW thickness and the lattice strain across the DW.

Like reported in refs. ^50,51^, we determine the DW thickness by measuring the [010]-projected off-centre Fe displacements. Therefore, the DW thickness is represented as the projected correlation length along which Fe displacements are perturbed due to the spontaneous polarization reversal at the DW. Figure 2e,f shows these displacements across the DWs in the pristine and quenched samples along the [100] direction (parallel to the DW plane) and the [001] direction (perpendicular to the DW plane). Small variations in the [001] displacements and a change in the sign of the [100] displacements observed across the two DWs support their head-to-tail configuration^50^. By defining the region in which the [100] Fe displacements deviate from the nearly invariable displacement measured inside the two adjacent domains (see Fig. 2e, f and the delineated dashed red boxes), we determine the DW thickness of ~6 and ~3 unit cells for the DW in the pristine and quenched sample, respectively.

A similar thickness reduction as observed after quenching was also found in the DW switched by the electric field (Fig. 1b, e). In this case, the switched DW, which is depleted of Bi vacancies, is ~3 unit cells thick (see dashed orange box in Fig. 1e), while the pristine DW, with accumulated Bi vacancies, is much thicker, i.e. ~9 unit cells (see dashed orange box in Fig. 1b). Considering that both the electric field and the quenching lead to the same depletion of Bi vacancies from the DW (Figs. 1f and 2d), it is suggested that these vacancies represent a necessary element for the DW broadening in the pristine sample. In fact, isolated Fe^4+^ defects accumulated in the switched DW do not lead to an extensively broadened DW (~3 unit cell, dashed orange box in Fig. 1e).

The lattice-strain distribution across the DW in the pristine and quenched sample was determined by extracting the unit-cell distortion angle along the [001] direction (*ϕ*_c_; Fig. 2g, h), as reported by Wang et al.^50^. The reason for choosing this lattice-strain representation is that the distortion angle *ϕ*_c_ is related to the Bi-sublattice shear strain, which was predicted by DFT to be intrinsic to all the three types of DWs in rhombohedral BiFeO_3_^51^. Note in Supplementary Fig. 8 that the lattice-distortion angle along the [100] direction, *ϕ*_a_, is close to zero throughout the analysed regions and thus much lower than *ϕ*_c_, meaning that the lattice is indeed subjected to a shear-like distortion predominantly in the [100] direction (*ϕ*_c_»*ϕ*_a_). Consistent with the DFT analysis of the defect-free DWs^51^, Bi shearing in the [100] direction is also observed in our quenched BiFeO_3_ in which the DW is depleted of defects (see red lines in Fig. 2b connecting Bi atoms inside the DW). This shear deformation is reflected in *ϕ*_c_ peaking at ~6° in the centre of the wall (Fig. 2h). Although with smaller *ϕ*_c_, this lattice strain expands several nanometres outside the DW region (see Supplementary Fig. 8 showing an extended view of the same region as that shown in Fig. 2h). This result is not unexpected considering that the strain fields arising from the DWs may extend relatively far (micrometres) into the domains, as shown for BaTiO_3_^52,53^. Interestingly, in BiFeO_3_ thin films, similar long-range strain fields were also found to arise from dislocations^54^.

The presence of defects at the wall has a measurable effect on the lattice-strain distribution inside and outside the DW region. Compared to the DW depleted of defects (quenched), in the pristine sample an enhanced shear strain is clearly observed (see red lines in Fig. 2a and compare with those shown in Fig. 2b), corresponding to the maximum *ϕ*_c_ of ~8° confined to a thinner region inside the wall (Fig. 2g). This lattice distortion coincides with the location where the concentration of Bi vacancies is the highest (compare red contour region in Fig. 2g with reddish circles in Fig. 2c). The results reveal a previously unreported role of Bi vacancies in the Bi shearing. Interestingly, in addition of being maximized in the region with a high Bi-vacancy concentration, the lattice strain also abruptly reduces in the surrounding area (see dark-blue contour region, surrounding the red area, inside and outside the DW in Fig. 2g). This strain distribution appears to be different to that in the DW with depleted defects where the lattice strain tends to expand further from the DW (Fig. 2h and Supplementary Fig. 8). To obtain a quantitative picture of the lattice distortion confined to the DW region, we determine a distortion angle *ϕ*_c_ that is spatially averaged over the DW area (Supplementary Fig. 8). This average angle is found to be lower in the pristine sample (1.9° per unit cell) than that in the quenched sample (2.9° per unit cell), indicating that, on average, the shear strain inside the DW is released by the accumulated defects.

## Discussion

**Fig. 4:**
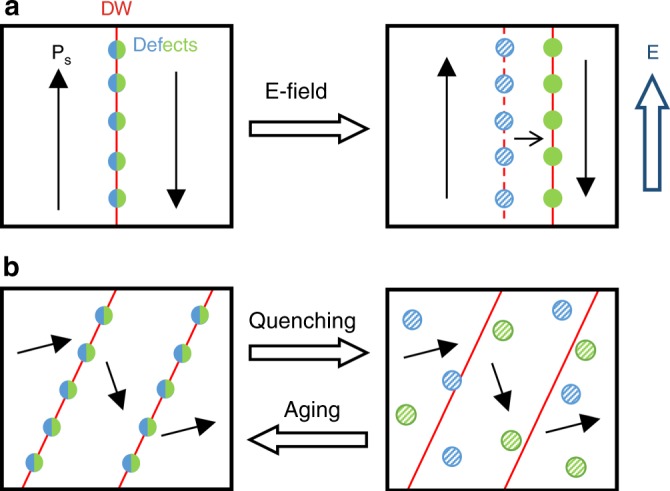
Effect of electric field and thermal treatment on defect state. Simplified schematic representation of the effect of **a** electric-field (E) and **b** quenching and aging on the defect location. The red lines, black arrows and colored circles indicate the domain walls (DWs), spontaneous polarization (P_S_) and point defects, respectively. The blue and green circles represent Bi vacancies and electron holes (Fe^4+^ states), respectively (same colour coding is used for the half-coloured circles). For simplicity, the oxygen vacancies are not considered. Locations of defects represented by full circles were experimentally confirmed by STEM, while locations of the defects represented by patterned circles are assumed based on ion-mobility (panel **a**, right box) and quenching concepts (panel **b**, right box). The dashed red line in the right box of panel **a** represent the initial DW position before field application; here, the patterned circles, representing Bi vacancies, were drawn on this line for illustrative purposes, however, short-range Bi-vacancy migration cannot be completely excluded. The scheme shown in panel **a** corresponds to the DW pinning model developed by Postnikov et al.^55^; see main text for further details. The choice of the domain-wall type (180° and non-180°) is arbitrary. Note that in the real scenario due to the thermal excursion above *T*_c_, the domain structure after quenching is most likely not the same; in panel **b** the same DWs in the left and right boxes are thus drawn for simplicity and for only purpose to represent the order and disordered defect state in aged and quenched samples, respectively. Due to the high quenching temperature (900 °C), the defects in the quenched state are assumed dissociated.

By revealing the tendency of electron holes (Fe^4+^) to couple to DWs under an applied field, the ex situ electric-field STEM experiment (Fig. 1) confirms on the atomic scale the model proposed by Postnikov et al.^55^ in 1970s. The model assumes DW pinning by accumulated defects with depinning occurring under the application of subswitching fields for prolonged time, during which the defects can migrate along with the moving DW (Fig. 4a). The model thus implies that the irreversible DW displacements become strongly time dependent and should thus occur at low driving-field frequencies. This scenario is supported by the evidence of moving conductive DWs in poled polycrystalline BiFeO_3_ at Hz and sub-Hz electric-field frequencies^13,56^. We point out that the inability of the low-diffusive Bi vacancies to follow the DW displacements suggests that in poled samples the DWs will mostly accumulate the mobile electron holes (such poled case is represented by the right box in Fig. 4a). Therefore, the pinning mechanism related to the coupled DW-holes dynamics is expected to play the key role in the piezoelectric response of poled BiFeO_3_, while the Bi-vacancy mediated pinning likely have a role in unpoled samples (represented by the left box in Fig. 4a) when excited by weak fields.

The DW-hole dynamic coupling implies that the pinning is mediated by individual defects, which is fundamentally different from the pinning mediated by reorientable defect complexes as the mechanism widely accepted for hard PZT and BaTiO_3_^24–27,57^. This agrees with recent first-principles calculations showing that Bi vacancies and electron holes in BiFeO_3_ do not have a tendency for close association with the holes being weakly trapped by the crystal lattice^44^. A similar scenario of weakly trapped holes was predicted by DFT in PbTiO_3_ where it was shown that the trapping state may be altered when the holes reside inside the DW region^58^. Therefore, the available first-principles calculations for BiFeO_3_^44^ indicate that electron holes can be easily separated from Bi vacancies under an applied electric field, as it can be deduced from the results presented in Fig. 1. In contrast to these results, Stolichnov et al.^22^ reported on a decoupling between conductive paths and switched DWs in BiFeO_3_ thin films. These two cases strongly support the idea that different accumulated defects with rather different mobilities and tendencies for association can control the DW conductivity and dynamics in BiFeO_3_ polycrystals and thin films.

An interesting question arising is why the positively charged electron holes ($Fe_{Fe}^ \cdot$ in Vink-Kröger notation) follow the DW displacements after separating from the negatively charged Bi vacancies ($V_{Bi}^{\prime\prime \prime }$, if assumed fully ionized), considering than in this case the local defect-charge electroneutrality is broken? A possible explanation could be the driving forces for the defect accumulation at DWs. One of the reasons for such an accumulation is the screening of uncompensated polarization charges at the walls^3,20^. Considering that our results reveal the reaccumulation of electron holes (Fe^4+^) at the switched DW, which, unlike the Bi vacancies, are rather mobile, we may presume that the defect accumulation at DWs under electric field is controlled by the defect mobility. The presence of Fe^4+^ at DWs after switching indicates that these accumulated defects probably still contribute to the screening of polarization charges at the DWs. This opens up interesting and complex questions on the internal electrostatic and strain states at DWs under applied external electric or even stress fields, which could be resolved by first-principles computations.

According to the defect-chemistry model proposed for BiFeO_3_^39,41^, the samples analysed in this study should contain oxygen vacancies. Semi-quantitative EELS OK-edge analyses on the investigated BiFeO_3_ samples indicate no oxygen-vacancy accumulation at the DWs above the uncertainty limit of a few at.% (Supplementary Fig. 9). This concentration is much lower than that of accumulated Bi vacancies (~30 at.%; see ref. ^21^). We can thus infer that the head-to-tail DWs analysed in this study predominantly accumulate Bi vacancies and electron-hole defects. This does not rule out, however, the likely pinning effects mediated by oxygen vacancies, which might be located inside the domains or other sample regions.

We show on the atomic level that the originally accumulated defects at the DWs deplete from these interfaces by quenching the samples from above *T*_c_, while they reaccumulate in the reverse process of aging (Fig. 4b). The aging strongly affects the electron-hole-mediated DW conductivity (Fig. 3). The results thus imply that, in addition to the previously discussed thermodynamic variables, such as the partial pressure of oxygen and the temperature^17,19,21^, defining the type and concentration of defects, also the kinetic parameters, e.g. the cooling rate during the material synthesis, should be considered in light of the reproducibility of the DW conductivity, which is a key aspect that must be solved prior to introducing conductive DWs into nano-devices.

It has been suggested that the accumulation of defects at the DWs should make the DWs thicker^2,47,59^. The data presented here for BiFeO_3_ support this hypothesis (Figs. 1,2) and reveal that Bi vacancies have a key role in broadening the 109° DWs. These observations experimentally validate the phenomenological model developed for PbTiO_3_^59^, in which defect clustering was found to broaden the wall above its intrinsic, defect-free thickness (~1 unit cell). Broadening effects due to DW pinning by defects has also been theoretically discussed by first principles for 90° DWs in PbTiO_3_^60^.

The Bi-sublattice shear strain at DWs in BiFeO_3_^51^ (Fig. 2) can in principle attract defects by coupling to the strain inherently related to the defects themselves (the so-called “chemical strain”)^61^. In fact, DFT studies on a 90° DW in PbTiO_3_ showed that the formation energy of oxygen vacancies is lowered in proportion to the increasing magnitude of the shear strain at the wall^62^. Another theoretical study on a 180° DW in PbTiO_3_ also predicts a release of the tensile stresses at DWs due to the accumulation of ionic vacancies^58^. A similar argument of strain at neutral DWs acting as a driving force for defect accumulation/depletion was used in theoretical studies on rare-earth manganites^63^. In our study, we confirm that the defects spontaneously accumulate at the DWs during aging. With the Bi vacancies and electron holes accumulated, the lattice shear strain inside and beyond the DW region is redistributed (Fig. 2) with the net result of being released on average inside the DW. Therefore, the chemical strain should be more extensively considered in future theoretical and experimental studies as it could provide new means for defect ordering at the DWs, beyond the extensively discussed electrostatic concepts. We finally note that this idea should be applied on a broader basis, considering that also long-range strain fields arising from dislocations can act as a driving force for atom diffusion as recently shown in Pb(Zr,Ti)O_3_ thin films^64^.

In summary, using atomic-resolution microscopy and PFM/c-AFM analyses we provide atomistic details of the coupling between DWs and charged point defects in polycrystalline BiFeO_3_. The results could be useful in the engineering of DW conductivity and dynamics for future DW-based devices.

## Methods

### Sample preparation

BiFeO_3_ ceramic samples were prepared from mechanochemically activated powders. Powder compacts were sintered at 760 °C for 6 h with a heating and cooling rate of 5 °C/min, resulting in a relative geometrical density of ~93%. Details of the processing procedure and microstructural properties of the as-prepared BiFeO_3_ samples are reported in ref. ^65^, while structural data using synchrotron X-ray diffraction are provided in ref. ^66^. Like reported in our previous studies^13,21^, the pristine sample for PFM and c-AFM analyses was additionally thermally relaxed with an annealing excursion at 840 °C (zero hold time) using the same heating and cooling rate of 5 °C/min as during the sintering. To induce defect disorder, the pristine samples were rapidly heated (within 2 min) to above *T*_c_, i.e. to 900 °C, kept for 5 min at this temperature and then quenched by dropping them either into pre-heated water (70 °C) or on a copper block, like reported in refs. ^32,65^ (the results are shown for water-quenched samples). Note that the two quenching procedures resulted in qualitatively the same effects on the *P*–*E* hysteresis (Supplementary Fig. 6). Aging and thus defect reordering was performed by annealing copper-quenched samples at 760 °C for 6 h using a heating rate of 5 °C/min and a slow cooling rate of 0.5 °C/min to allow defect diffusion (see ref. ^32^). Finally, the surfaces of all the samples after sintering, quenching and aging were ground in order to remove ~100 μm of the surface layers.

### Electron microscopy characterization

Samples for STEM analyses were prepared by standard methods described in ref. ^21^. STEM studies were performed using a probe Cs-corrected Jeol ARM 200 CF microscope operated at 200 kV. EELS measurements were carried out using a GATAN Quantum ER Dual EELS spectrometer. Data elaboration of the HAADF images (see Supplementary Fig. 10) and EELS analyses, including Fe displacement and Bi intensity mapping, are reported in ref. ^21^; identification of the Fe oxidation state using EELS is additionally explained in Supplementary Fig. 2. For the ex situ electric-field experiment, a sample prepared for standard STEM characterization was first electroded on two opposite sides using a flash-drying Ag paste and then exposed to 700 V for 10 min at room temperature using a DC power supply (Spellman SL150). A photograph of the sample is provided in Supplementary Fig. 1.

### PFM and c-AFM characterization

The sample surfaces for the PFM and c-AFM characterization were prepared by standard metallographic procedures explained in detail in ref. ^13^. PFM and c-AFM imaging was performed using a Molecular Force Probe 3D AFM from Asylum Research. A tetrahedrally shaped silicone AFM tip with a silicone cantilever, both coated with Ti/Ir (Asylec, AtomicForce F&E GmBH), was used for analyses. Out-of-plane PFM imaging was performed in Dual a.c. Resonance Tracking (DART) mode using 8 V of a.c. voltage at the tip. c-AFM imaging was carried out by applying to the tip a d.c. bias voltage in the range from 7 to 17 V (c-AFM maps in Fig. 3 were acquired at 13 V d.c.). The scan frequency and scan-points-per-line were, in all cases, 0.8 Hz and 256, respectively.

## Supplementary information

Supplementary Information

## Data Availability

All the data supporting the findings in this study are available in the manuscript and in Supplementary Information. Further data and methods are available from the corresponding authors upon request.
