# Supplementary material for: Domain-wall pinning and defect ordering in BiFeO3 probed on the atomic and nanoscale
Source: Nat Commun. 2020 Apr 9;11:1762. doi: 10.1038/s41467-020-15595-0 (PMC7145836; doi:10.1038/s41467-020-15595-0)
Supplement: Supplementary file 1 — Supplementary Information [file 41467_2020_15595_MOESM1_ESM.pdf]

SUPPLEMENTARY INFORMATION

Domain-wall pinning and defect ordering in $BiFeO_3$ probed on the atomic and nanoscale

Andreja Bencan[1,2], Goran Drazic[2,3], Hana Ursic[1,2], Maja Makarovic[1,2] Matej Komelj[4] & Tadej Rojac[*1,2]

[1]*Electronic Ceramics Department, Jozef Stefan Institute, 1000 Ljubljana, Slovenia*

[2]*Jozef Stefan International Postgraduate School, 1000 Ljubljana, Slovenia*

[3]*Department of Materials Chemistry, National Institute of Chemistry, 1000 Ljubljana, Slovenia*

[4]*Department for Nanostructured Materials, Jozef Stefan Institute, 1000 Ljubljana, Slovenia*

*Corresponding author (email: tadej.rojac@ijs.si)

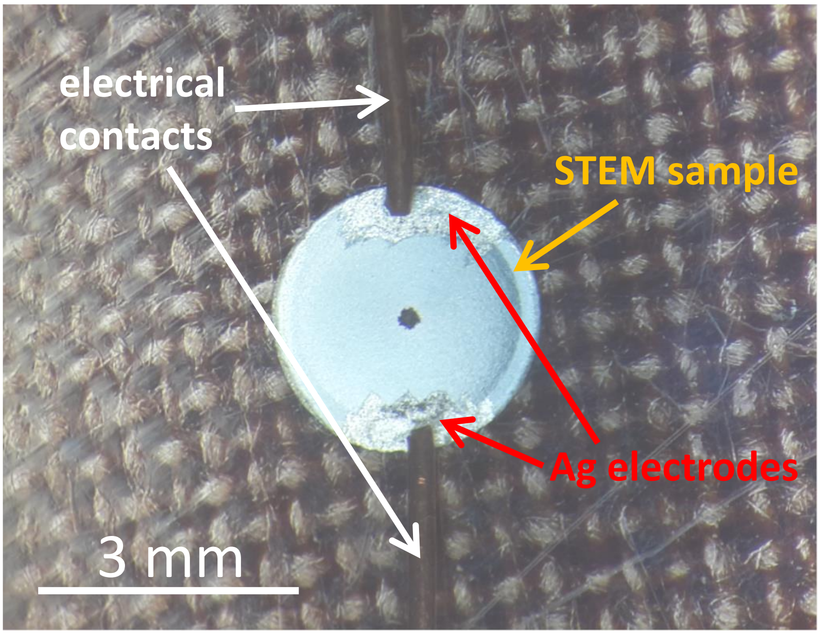

Supplementary Figure 1. Sample for ex-situ electric-field scanning-transmission electron microscopy (STEM) analysis. Optical microscope image of the $BiFeO_3$ sample for STEM analysis showing the Ag electrodes painted at opposite peripheral sides of the circular sample and electrical contacts for the field application. The sample was analysed in the same region close to the central ion-milled circular part before and after application of the electric field. Further details regarding sample preparation and electric-field conditions are reported in Methods.

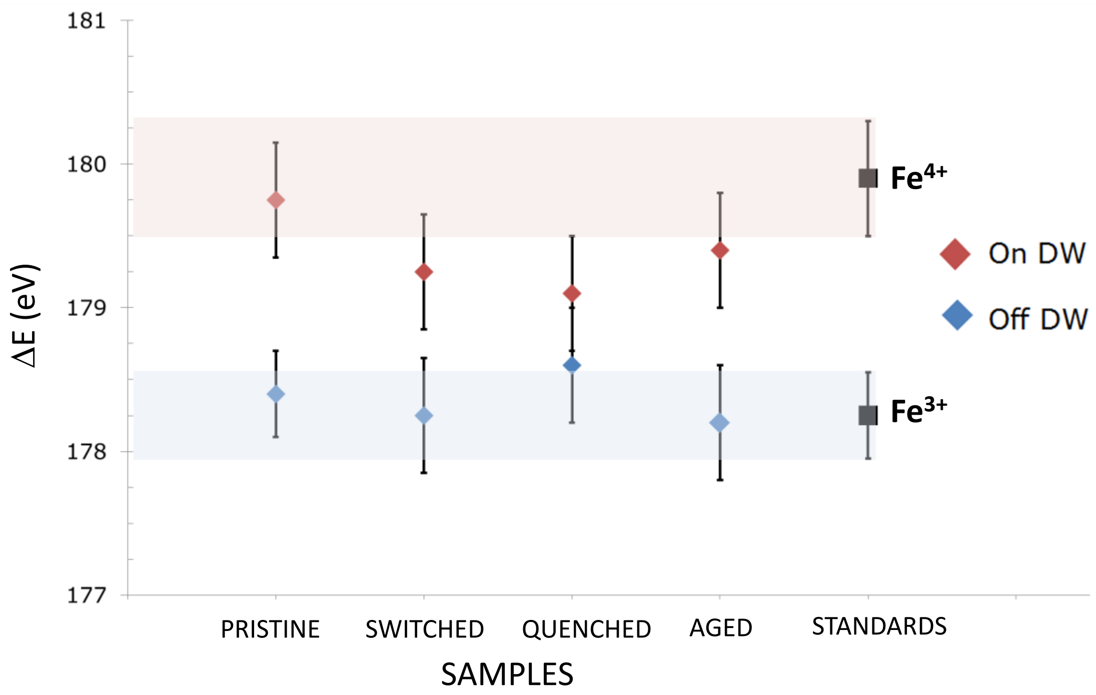

Supplementary Figure 2. Determination of oxidation state of Fe ions inside domain-wall (DW) regions in $BiFeO_3$ samples using electron energy-loss spectroscopy (EELS) analysis. The plot shows the experimental values of the energy onset difference (ΔE) between the O-K and Fe-L_3 edges determined on (red diamonds) and off (blue diamonds) DW regions in pristine, switched, quenched and aged $BiFeO_3$ sample. The "switched" sample refers to the ex-situ electric-field experiment shown in Fig. 1 in the main paper and Supplementary Fig. 1, while the aged sample refers to the quenched $BiFeO_3$ that was additionally annealed and cooled with a slow rate (see Methods). For comparison, the ΔE values obtained on standard materials consisting of Fe^{4+} ($BaFeO_3$) and Fe^{3+} states (Fe_2O_3) are added (black squares). Red and blue bands as well as error bars represent measurement errors defined by the standard deviation of measurements determined either on standard materials (bands) or $BiFeO_3$ samples (bars). Note that the ΔE values were determined as an average of measurements on at least three different regions on and off the DW in respective $BiFeO_3$ samples. With the exception of the quenched sample, in all other samples the difference between the average ΔE values determined on and off the DW is significant (at least 1eV), indicating the presence of Fe^{4+} at the DWs. In the quenched sample, on the other hand, this ΔE difference (0.5 eV) is within the measurement error, suggesting the absence or strongly reduced concentration of Fe^{4+} at the walls. Further details of the energy difference method applied to $BiFeO_3$ and EELS analyses on standard materials are reported in ref.[1].

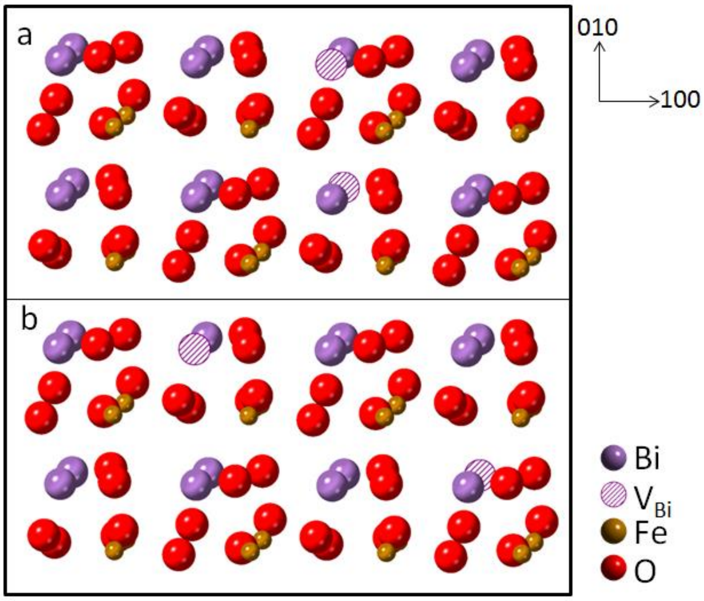

Supplementary Figure 3. ***Ab-initio*** **total-energy calculations in $BiFeO_3$ with accumulated and dispersed Bi vacancies.** *R3c* structural model of $BiFeO_3$ (ICSD-75324) used for DFT calculations with Bi vacancies **a** accumulated in one (100) Bi plane and **b** dispersed within two unit cells (see patterned circles in the two models indicating Bi vacancies). The calculations were carried out within the framework of the density-functional theory (DFT) by applying the Quantum Espresso[2] code. The interaction between the core and valence electrons was described by the projector-augmented-wave (PAW) pseudopotentials of the Troullier-Martins-type[3], whereas the exchange-correlation effects were treated within the generalized-gradient approximation (GGA)[4]. The wave functions and the charge densities were expanded in the plane waves with the cut-off parameters 680 eV and 6800 eV, respectively, and the 2x4x4 mesh was used for the Brillouin zone integration. Both structures were optimized by finding the equilibrium lattice parameters and minimizing the interatomic forces. The criterion for the self-consistency was the energy difference between two subsequent iterations being less than 10^{-7} eV, which is much lower than the calculated energy difference of 1.32 eV between the two structures (see text below for further details).

The results shown in Fig. 1 in the main paper suggest that Bi vacancy do not have a tendency to migrate along the moving DW under applied electric field as no Bi vacancy accumulation was observed in the switched DW area. In the first approximation and for simplicity reasons, we elaborate the case in the absence of electric field by hypothesizing a situation in which the Bi vacancies, originally accumulated at the DW, remain in the same lattice positions after this DW is displaced. In order to evaluate whether these accumulated Bi vacancies would have a tendency to disperse (i.e., to disorder), we performed a*b-initio* DFT calculations of the total energies E_a and E_b for two different structures in $BiFeO_3$ containing either accumulated Bi vacancies in one (100) Bi plane (supplementary Fig. 3a) or dispersed Bi

vacancies within two unit cells (supplementary Fig. 3b). The calculated energy difference is E_a–E_b=0.66 eV per Bi vacancy. The results confirm that the energy of the dispersed Bi vacancies (E_b) is lower than that of accumulated vacancies (E_a), suggesting the tendency of the Bi vacancies to disperse from their initial accumulated positions after isolating from the DW (due to the DW being displaced into an another position). Despite this driving force, however, we point out that the process of dispersion of the initially accumulated Bi vacancies will be strongly controlled by the diffusivity of these vacancies. In perovskites, A-site vacancies are well known to exhibit a much lower diffusivity than the O vacancies[5]. This is, for example, supported by recent molecular dynamics simulations in $(Na_{0.5}Bi_{0.5})TiO_3$ perovskite, which confirm a much higher activation barrier for Bi^{3+} migration via the vacancy mechanism (between 5 and 9 eV, depending on the particular migration path) compared to that of O^{2-} (<0.8 eV)[6].

In the absence of diffusion data in $BiFeO_3$, for estimation purposes, we use the experimentally determined diffusion coefficients for Bi migration in V_2O_5-doped Bi_2O_3 reported by Palkar *et al.*[7]. As discussed in that study, the V donor dopant results in the formation of compensating Bi vacancies, thus the reported data should closely reflect the self-diffusion of Bi^{3+} in the presence of Bi vacancies. The diffusion coefficients, extrapolated for our case to low temperatures (i.e., between 20°C and 100°C), are in the range D~10^{-22}–10^{-18} cm^2 s^{-1}. Using the same Fick's second law solution of the diffusion model reported in that paper, we can estimate the average diffusion distance as $\bar{x} = \sqrt{2.77 \cdot D \cdot t}$ where D is the diffusion coefficient and t is the diffusion time ($\bar{x}$ corresponds to the diffusion distance where the relative concentration of the diffusing species is 0.5). For a diffusion time of 1 hour, the extrapolated D coefficients result in $\bar{x}$ in the range between 0.01 and 1 nm, thus suggesting diffusion over a maximum distance not longer than ~2 unit cells.

While the simplified analysis proposed here does not take into account the electric field effects on the Bi-vacancy migration, the data still show that while the configuration of the accumulated Bi vacancies is energetically unfavorable in the absence of the DW (being displaced by the field), the low diffusivity of the Bi vacancies prevents them to migrate over distances longer than few lattice sites, as proposed in the main paper.

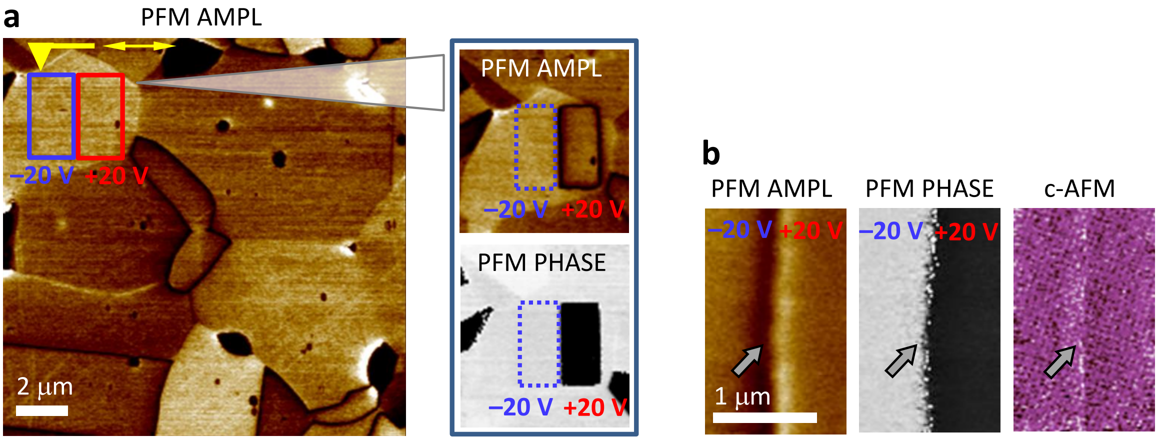

Supplementary Figure 4. Domain-wall lithography in polycrystalline $BiFeO_3$. a Illustration of piezo-response force microscopy (PFM) litographic procedure in polycrystalline $BiFeO_3$ matrix. The PFM out-of-plane (OP) amplitude image shows a larger area of the pristine sample with marked rectangular regions of approximate dimensions 5 x 2 µm, which were scanned with 0.8 Hz of frequency by alternating –20 V and +20 V of d.c. bias on the tip (see pictures of the tip and its scanning direction above the rectangles). Insets show PFM OP amplitude and phase images of the switched domains inside the grain. The opposite direction of the out-of-plane polarization component in the two switched rectangles is evident from the white/black phase contrast. **b** PFM OP amplitude and phase enlarged-view images of the written DW (arrow) along with the conductive atomic-force microscopy (c-AFM) map. The enhanced current signal detected at the wall position confirms the conductive character of the litographed DW. The results are consistent with the observed re-accumulation of electron holes (Fe^{4+} states) in the switched DW region shown in Fig. 1 of the main paper, supporting the link between the accumulated p-type carriers and the DW conductivity.

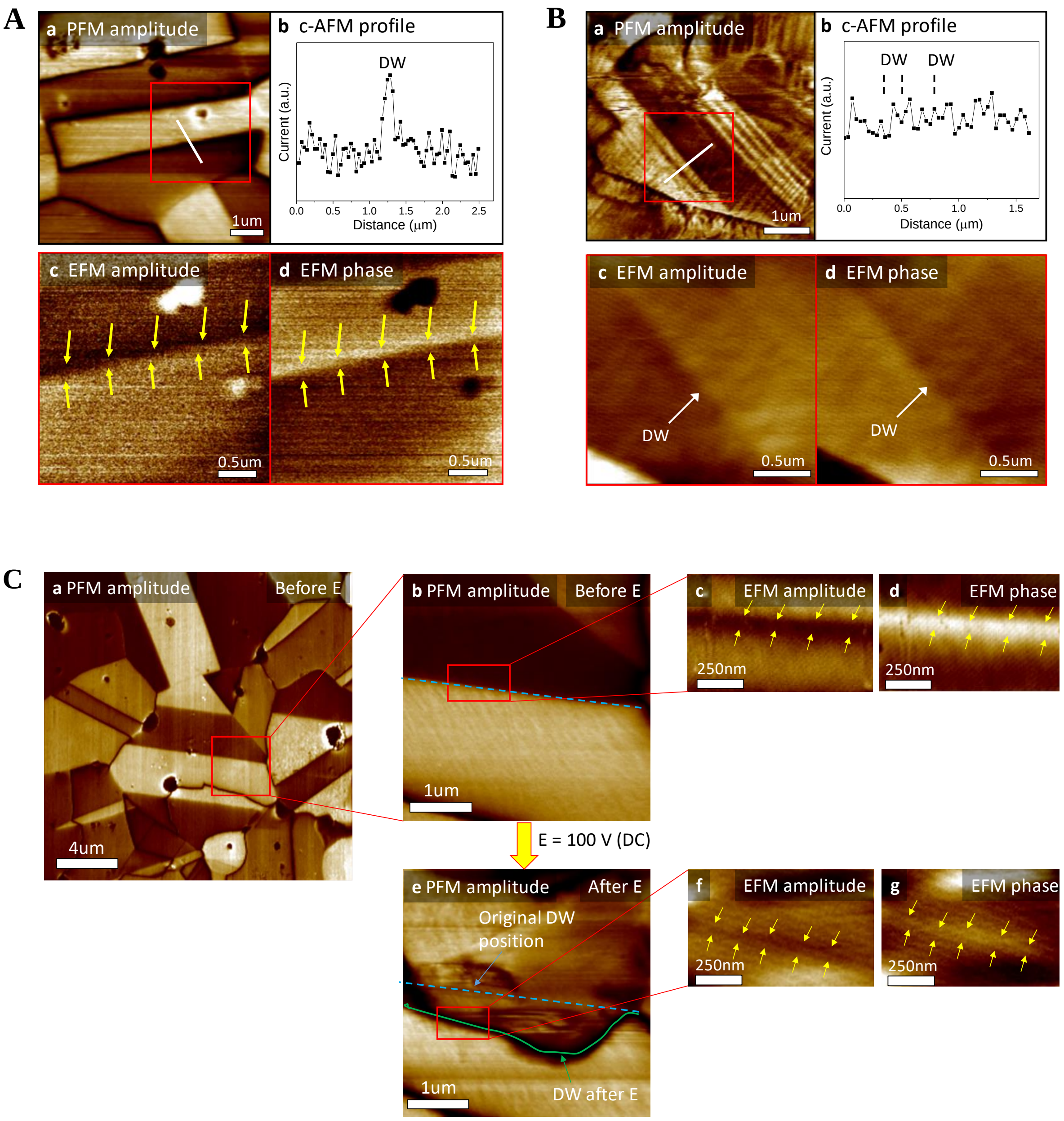

Supplementary Figure 5. Electrostatic force microscopy (EFM) imaging of DWs in $BiFeO_3$ and "soft" Pb(Zr,Ti)O_3 (PZT). Panel **A** and **B** show EFM analyses of DW regions in pristine $BiFeO_3$ and "soft" PZT, respectively, while panel **C** shows EFM analyses on DWs in $BiFeO_3$ before and after switching a region with the AFM tip. **Aa** PFM OP image of the analyzed region in $BiFeO_3$. **Ab** Electric-current (c-AFM) profile measured along the white line noted in panel Aa; the position of the DW is shown on the profile. **Ac** EFM amplitude and **Ad** EFM phase image of the region indicated with a red box in panel Aa. The yellow arrows show the reduced amplitude (dark contrast) and increased phase (bright contrast) of the EFM signal measured at the DW position. **B(a–d)** Same as A(a–d) but measured on a 2 mol%-Nb-doped morphotropic "soft" PZT with the nominal composition $Pb(Zr_{0.53}Ti_{0.47})O_3$. **Ca** PFM OP image of the analyzed region in $BiFeO_3$. **Cb** Enlarged PFM amplitude image corresponding to the red box in panel Ca; the DW is marked with a dashed blue line. **Cc** EFM amplitude and **Cd** EFM phase of the region marked with a red box in panel Cb. **Ce** PFM amplitude image of the same

region as shown in panel IIIb, but after switching it by scanning with the tip biased with +100 V DC. The dashed blue line and full green line denote the original DW position before switching and the new DW position after switching with the AFM tip, respectively. **Cf** EFM amplitude and **Cg** EFM phase of the switched DW with the yellow arrow indicating the persistence of the dark and bright EFM contrast in the amplitude and phase, respectively, after switching the DW (compare with the EFM signal at the DW before switching, i.e., yellow arrows in panels Cc,d). EFM imaging was performed using the "Nap pass" mode with a 40 nm lift-off distance of the AFM tip above the sample. During imaging the tip was held at constant DC bias of 11 V, 9 V and 6 V for the cases shown in panel A, B and C, respectively.

To probe the electrostatic signal at DWs, we compare the EFM analysis on pristine $BiFeO_3$ (supplementary Fig. 5A) with that of a "soft" morphotropic PZT (supplementary Fig. 5B). The purpose was to analyze the EFM signal at conductive DWs in $BiFeO_3$ and compare it with the signal at the DWs in "soft" PZT, which do not show a clear conductive character (a comparative study between $BiFeO_3$ and "soft" PZT in terms of DW conduction and macroscopic piezoelectric response is reported in Ref.[8]).

In the case of $BiFeO_3$, the analyzed DW, which shows a conductive behavior (see c-AFM profile in supplementary Fig. 5Ab), exhibit an anomaly in the EFM signal, reflected by a darker contrast in the EFM amplitude (see arrows in supplementary Fig. 5Ac) and brighter contrast in the EFM phase (see arrows in supplementary Fig. 5Ad), relative to these two signals measured in the adjacent domains. In contrast, no such clear anomaly is observed in the EFM signals at the DW of "soft" PZT (supplementary Fig. 5Bc,d), where an enhanced electric current at the DW was not detected (supplementary Fig. 5Bb). Although the origins of the electrostatic signals at the DWs in these materials might be complex considering that they can be in principle affected by both mobile and static charges[9,10], the different electrostatic response of the DWs in $BiFeO_3$ and "soft" PZT could be in part related to the conductive nature of the DWs in the ferrite; this is because the presence of mobile charges at DWs may contribute to the screening of the charges on the biased tip, eventually affecting the electrostatic response[9,10].

To further verify whether the electrostatic configuration at the DW is altered after switching the wall with an electric field, we performed and experiment where a region in $BiFeO_3$ (supplementary Fig. 5Ca) was analyzed in terms of PFM and EFM before (supplementary Fig. 5Cb–d) and after the switching process (supplementary Fig. 5Ce–g). The DW before switching confirms the anomaly in the EFM signal as explained in the previous case (see supplementary Fig. 5Cc,d and compare with Fig.5Ac,d). Switching the area with the biased tip clearly displaced the DW in a new position (see green line in supplementary Fig. 5Ce where the dashed blue line indicates the DW positon before the switching). EFM analysis of this displaced DW, shown in supplementary Fig. 5Cf,g, qualitatively confirms the same anomaly in the EFM signal at the wall. Note that the dark EFM amplitude contrast and bright EFM phase contrast at the switched DW is slightly reduced as compared to these signals at the DW before switching (see supplementary Fig. 5Cc,d); however, qualitatively, the same anomaly is still detected after the switching. The results therefore indicate that the electrostatic charge

configuration at the wall remains qualitatively the same after being switched with the electric field. Similarly, also the conductive state of the DW persists after switching the wall (see supplementary Fig. 4).

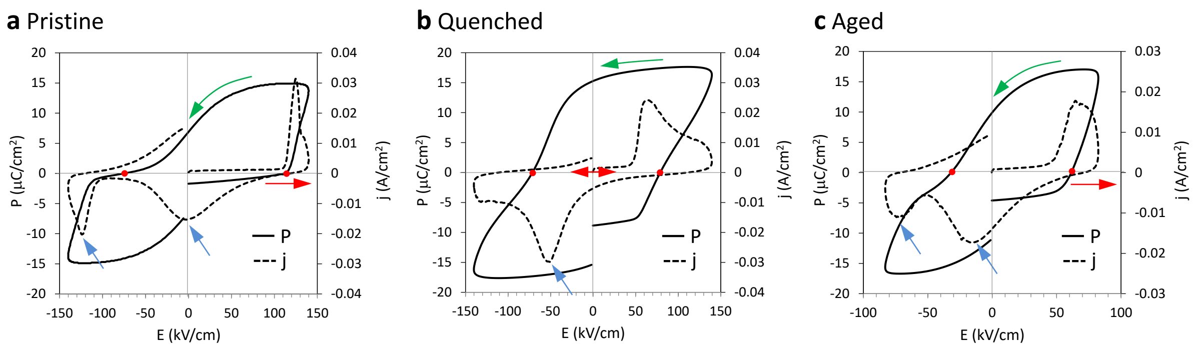

Supplementary Figure 6. Effects of quenching and aging on the macroscopic domain-switching behavior of $BiFeO_3$. Polarization–electric-field (*P*–*E*) and current-density–electric-field (*j*-*E*) hystereses loops of **a** pristine, **b** quenched and **c** aged sample. Note that the aged sample was quenched prior to aging.

The samples for measurements were prepared by standard methods including cutting the pellets, grinding them down to ~200 μm of thickness and Au electroding of the respective pellet surfaces. The measurements were performed using an aixACCT TF 2000 analyzer (aixACCT Systems GmbH, Aachen, Germany) by applying to the samples single sinusoidal voltage signals of 100 Hz of frequency with 10 kV/cm of field steps up to the maximum field (see also ref.[11]). Details regarding quenching and aging conditions are reported in Methods.

The *P*–*E* loop of the pristine sample is characterized by typical features commonly observed in the so-called "hard" ferroelectrics, such as acceptor-doped Pb(Zr,Ti)O_3 and $BaTiO_3$[12–15]: (i) pinched shape (reflected in the double switching-current peak; blue arrows), (ii) internal bias, often called "imprint", related to a shift of the loop along the electric-field axis (red dots and red arrow) and (iii) strong backswitching of domains during the field release (green arrow). All these features are fingerprints of strong pinning effects on DWs arising from the presence of ordered charged point defects. These "hardening" effects have been widely discussed for ceramic and thin-film $BiFeO_3$ and related compositions[16–22]. Comprehensive explanations of these phenomena on a general basis and from both microscopic and macroscopic perspective are reported in several review papers[23–26].

Upon quenching the sample in water from 900°C, the hysteresis-loop shape qualitatively changes. With respect to that in the pristine state, the loop after the quenching shows: (i) reduced pinching (only one switching-current peak can be clearly identified; blue arrow), (ii) reduced biasing (the loop is centered; red dots and red arrow) and (iii) reduced backswitching (smaller difference between maximum and remanent polarization; green arrow). Qualitatively, the exact same evolution of the hysteresis loop was observed by quenching a $BiFeO_3$ pellet on a large Cu block (not shown). This process, often referred to as the hysteresis relaxation[12], is in "hard" ferroelectrics, such as $BiFeO_3$, commonly attributed to the disordering of charged point defects[15,17,20]. The reason is that the material is cooled so rapidly from above the Curie temperature (~825°C for $BiFeO_3$[27]) that the defects are not given sufficient time for their ordering, e.g., by forming defect complexes or accumulating at DWs. On average, the pinning strength of the disordered defects is weaker than that provided by the ordered defects, hence the loop "relaxes", exhibiting depinching and de-biasing.[15,28]

The quenched disordered defect state can be reversed back to the ordered state by aging, e.g., by heating the sample to a sufficiently high temperature and by cooling it down to room temperature, this time slowly to allow the defect diffusion and thus ordering (e.g., in terms of the re-accumulation of the defects at the DWs). The high aging temperature (760°C; see Methods) was chosen in order to activate the Bi-vacancy diffusion while keeping it below the Curie temperature of $BiFeO_3$.

Consistent with previous studies on $BiFeO_3$[29], the hysteresis loop of the sample that was aged after quenching re-gained the pinched and biased shape (panel c), indirectly proving the re-ordering of the defects during aging. It is worth noting that, after aging, we did observe measurable changes in the mass of the pellets (i.e., above the uncertainty limit of ~0.05% relative) with only a minimal reduction of porosity (~1 %) and minimal increase in the average grain size (~0.2 % relative; results are not detailed here). This rules out microstructural features or Bi_2O_3 sublimation losses as the origins of the observed hysteresis-loop changes. To be noted is that the coercive field of the aged sample (red dots in panel c) is lower compared to that of the pristine and quenched sample. While the origin of this reduced macroscopic coercivity may be complex and multitude, we tentatively attribute it to a release of residual stresses, occurring during the slow cooling from the maximum aging temperature (0.5°C/min of cooling rate from 760 to 25°C), similarly as discussed for the surface-stress release in $BaTiO_3$ ceramics[30].

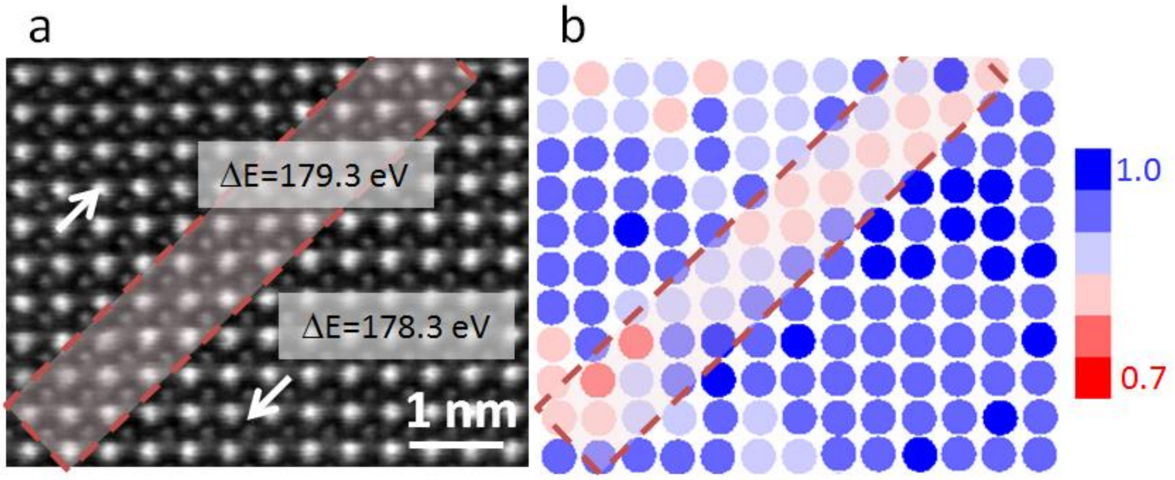

Supplementary Figure 7. Evidence of re-accumulation of defects (Bi vacancies and Fe^{4+}) in quenched $BiFeO_3$ sample after aging. a High-angle annular dark-field (HAADF) STEM image in [010] zone axis of a 180° DW (indicated by a dashed red box) in the aged $BiFeO_3$ sample with labelled ΔE between the O-K and Fe-L_3 edges. Arrows indicate the direction of the Fe displacements from the center of the Bi sublattice. **b** The corresponding normalized distribution map of the Bi-atom column intensities across the DW region. The reduction of the Bi-atom column intensities inside the DW (reddish circles) indicates the presence of Bi vacancies, consistent with their re-accumulation during aging of the quenched sample (considering that no Bi-vacancy accumulation was observed in the quenched sample prior to aging; see Fig. 2 in the main paper). Similarly, the 1 eV difference in ΔE measured on and off the DW (panel a) suggest the re-accumulation of Fe^{4+} at the wall (see also Supplementary Fig. 2 for statistical ΔE data on the aged sample).

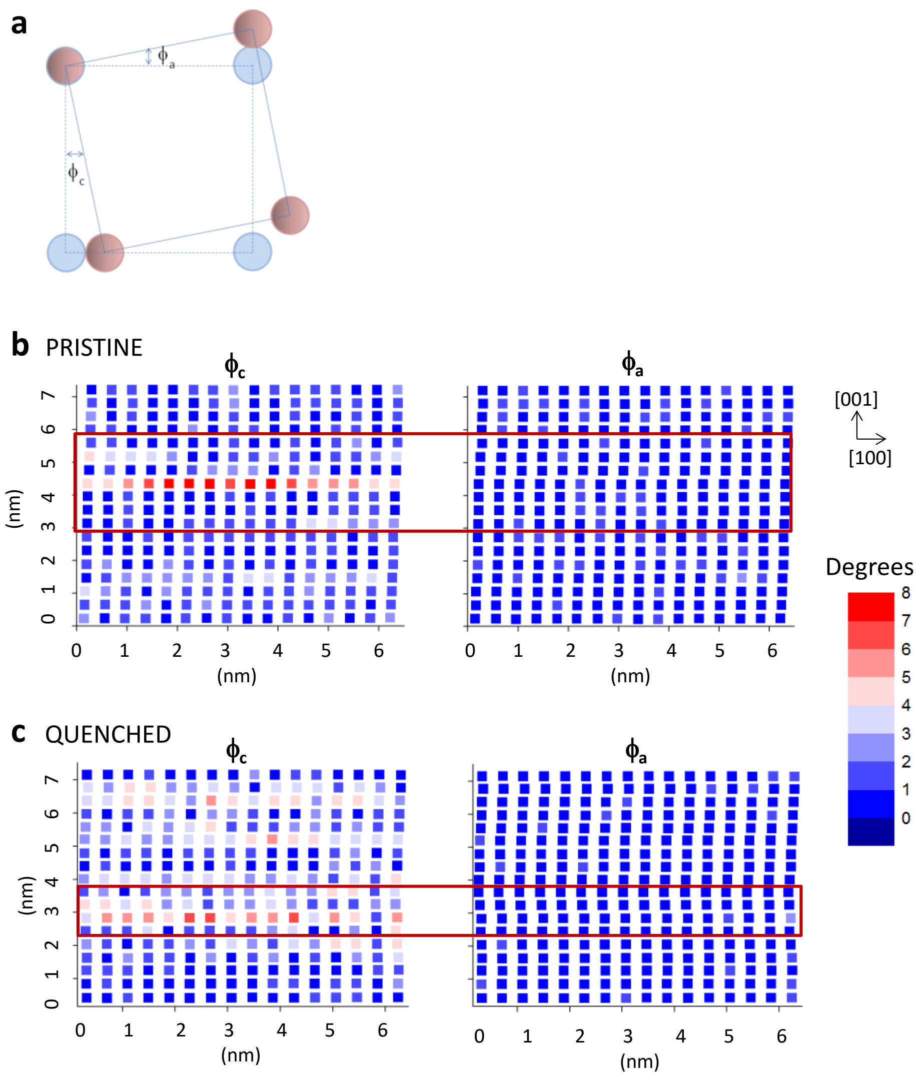

Supplementary Figure 8. Analysis of the lattice strain over larger sample areas surrounding the DWs in pristine and quenched $BiFeO_3$ samples. a Schematic of lattice-distortion angles. The lattice distortion is represented by a unit cell (red Bi atoms) rotated with respect to the ideal, undistorted unit cell (blue Bi atoms) where ϕ_a and ϕ_c are distortion angles along the [100] and [001] direction, respectively. These distortion angles are presented as strain maps for **b** pristine and **c** quenched $BiFeO_3$ sample. DW regions in the strain maps are marked with red boxes. The analysed regions in the two samples are the extended version of the regions shown in Fig. 2g,h in the main paper. Note that throughout the analysed regions $\phi_c >> \phi_a$, suggesting that the shear strain in the [100] direction predominates over that in the [001] direction. This strain arises due to the Bi shearing inside DWs[31] (see Fig. 2a,b in the main paper). Note that ϕ_c is largest inside the DW regions, however, in the quenched sample this strain expands further from the DW (light red and light blue squares outside the DW region in panel c), while in the pristine sample it is concentrated inside the DW region (red squares in panel b). In the pristine sample, the calculated average distortion angle ϕ_c and ϕ_a inside the DW region is 1.9 ± 0.9° and 0.6 ± 0.4° per unit cell, respectively. In the quenched sample, the average distortion angle ϕ_c and ϕ_a inside the DW region is 2.9 ± 1.1° and 0.4 ± 0.3° per unit cell, respectively.

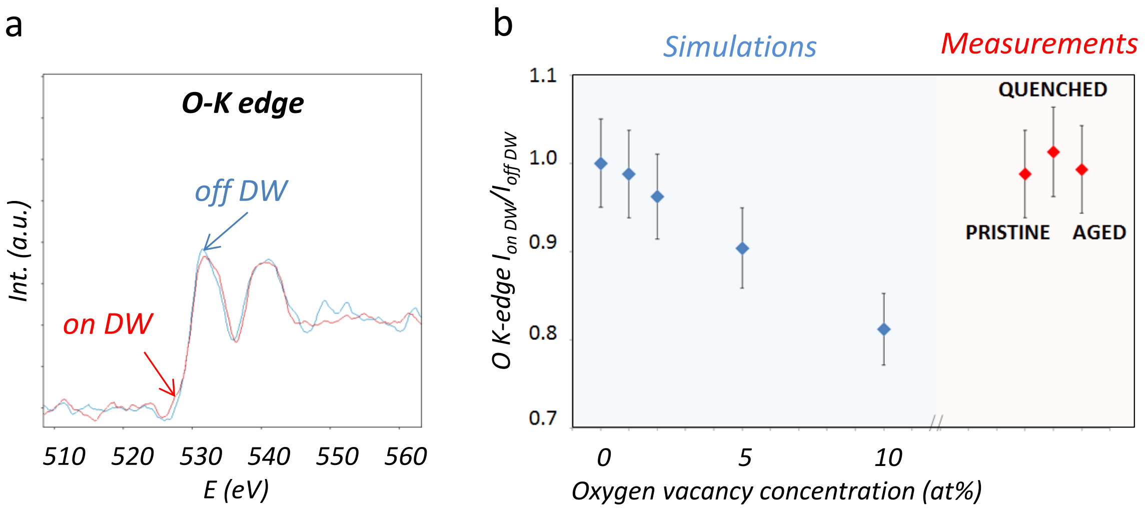

Supplementary Figure 9. Semi-quantitative EELS analysis of the O-K-edge intensities on and off the DWs in pristine, quenched and aged $BiFeO_3$ samples. a Example of EELS spectra (O-K edge) obtained on and off the DW in the pristine sample. Plural scattering was removed and both spectra were normalized with respect to the intensity of the Fe L_3-edge. The O-K-edge integral intensity was measured between 527 eV and 545 eV in both spectra and the ratio (i.e., $I_{on\ DW}/I_{off\ DW}$) was calculated. To semi-quantitatively evaluate the amount of oxygen vacancies in the DW regions, we simulated EELS spectra of $BiFeO_3$ with a different amount of vacancies (i.e., 0, 1, 2, 5 and 10 at%) using EELS Advisor simulation tool[32]. The simulated spectrum of $BiFeO_3$ without oxygen vacancies was used as an off-DW reference. Using the same approach as for the experimental spectra, we determined the O-K intensity integral ratio ($I_{on\ DW}/I_{off\ DW}$) as a function of the oxygen-vacancy concentration. These simulation data are plotted in panel **b** where the experimental integral ratios for the pristine, quenched and aged sample are added. Bars represent 5% measurement error determined by EELS analyses on several on and off DW regions in $BiFeO_3$ samples (these error bars are also added on the simulation data points). From the comparison between the experimental and simulated intensity ratios, we can conclude that the concentration of oxygen vacancies at the DWs in all three samples is less then ~5 at%. Therefore, using this method, at least 5 at% of oxygen vacancies should be present to be unambiguously determined.

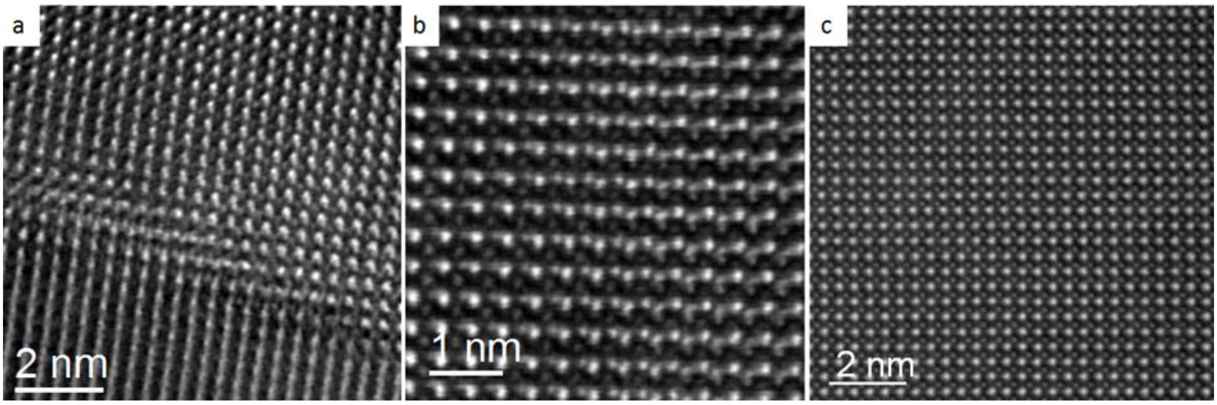

Supplementary Figure 10. Potential overlapping of various structural features inside examined areas with HAADF STEM. HAADF STEM images of **a** two overlapping $BiFeO_3$ crystallites along the electron-beam direction, **b** two overlapping ferroelectric domains in $BiFeO_3$ and **c** a DW area used in this study where no overlapping is present.

For quantitative structural and chemical analysis of DWs, high quality and drift-free HAADF STEM images were collected after stabilization of all instrumental parameters. The DW position was identified by local strains using low-angle annular dark-field (LAADF) imaging (see supplementary information, Fig.S3, of Ref.[1]). Potential overlapping of various structural features, such as grain boundaries and DWs, along the electron-beam direction inside examined areas were recognized as blurred or imperfect HAADF images. Such examples, which were avoided in the subsequent analyses, are shown above in supplementary Fig. 10a,b., while a typical example of a high-quality HAADF STEM image used for the analyses in this study is shown in supplementary Fig. 10c.

Supplementary References

1. Rojac, T. et al. Domain-wall conduction in ferroelectric $BiFeO_3$ controlled by accumulation of charged defects. *Nat. Mater.* **16**, 322–327 (2017).
2. Giannozzi, P. et al. QUANTUM ESPRESSO: a modular and open-source software project for quantum simulations of materials. *J. Phys.: Condens. Matter* **21**, 395502 (2009).
3. Troullier, N. & Martins, J. L. Efficient pseudopotentials for plane-wave calculations. *Phys. Rev. B* **43**, 1993–2006 (1991).
4. Perdew, J. P., Burke, K. & Ernzerhof, M. Generalized gradient approximation made simple. *Phys. Rev. Lett.* **77**, 3865–3868 (1996).
5. Uchino, K. *Ferroelectric devices* (Marcel Dekker, New York, 2000).
6. Zhang, H., Ramadan, A. H. H., De Souza, R. A. Atomistic simulations in sodium bismuth titanate (NBT) materials: towards superior oxide-ion conductors. *J. Mater. Chem. A* **6**, 9116–9123 (2018).
7. Palkar, G. D., Sitharamarao, D. N. & Dasgupta, A. K. Self-diffusion of bismuth in bismuth oxide. *Trans. Faraday Soc.* **59**, 2634–2638 (1963).
8. Rojac, T., Ursic, H., Bencan, A., Malic, B. & Damjanovic, D. Mobile domain walls as a bridge between nanoscale conductivity and macroscopic electromechanical response. *Adv. Funct. Mater.* **25**, 2099–2108 (2015).
9. Schaab, J. et al. Optimization of electronic domain-wall properties by aliovalent cation substitution. *Adv. Electr. Mater.* **2**, 1500195 (2016).
10. Schoenherr, P. et al. Observation of uncompensated bound charges at improper ferroelectric domain walls, *Nanolett.* **19**, 1659–1664 (2019).
11. Rojac, T., Kosec, M. & Damjanovic, D. Large electric-field induced strain in $BiFeO_3$ ceramics. *J. Am. Ceram. Soc.* **94**, 4108–4111 (2011).
12. Carl, K. & Härdtl, K. H. Electrical after-effects in $Pb(Zr,Ti)O_3$ ceramics. *Ferroelectrics* **17**, 473–486 (1978).
13. Lambeck, P. V. & Jonker, G. H. The nature of domain stabilization in ferroelectric perovskites. *J. Phys. Chem. Solids* **47**, 453–461 (1986).
14. Zhang, L. X. & Ren, X. In situ observation of reversible domain switching in aged Mn-doped $BaTiO_3$ crystals. *Phys. Rev. B* **71**, 174108 (2005).

15. Morozov, M. I. & Damjanovic, D. Hardening-softening transition in Fe-doped $Pb(Zr,Ti)O_3$ ceramics and evolution of the third harmonic of the polarization response. *J. Appl. Phys.* **104**, 034107 (2008).
16. Yuan, G. L., Yang, Y. & Or, S. W. Aging-induced double ferroelectric hysteresis loops in $BiFeO_3$ multiferroic ceramics. *Appl. Phys. Lett.* **91**, 122907 (2007).
17. Rojac, T., Kosec, M., Budic, B., Setter, N. & Damjanovic, D. Strong ferroelectric domain-wall pinning in $BiFeO_3$ ceramics. *J. Appl. Phys.* **108**, 074107 (2010).
18. Kim, A. Y. et al. Ferroelectric properties of $BiFeO_3$ ceramics sintered under low oxygen partial pressure. *J. Kor. Phys. Soc.* **60**, 83–87 (2012).
19. Lee, M. H. et al. Ferroelectric and piezoelectric properties of $BiFeO_3$-$BaTiO_3$ solid solution ceramics. *Ferroelectrics* **452**, 7–12 (2013).
20. Nam, H. et al. Influence of quenching temperature on piezoelectric and ferroelectrics properties in $BaTiO_3$-$Bi(Mg_{1/2}Ti_{1/2})O_3$-$BiFeO_3$ ceramics. *Ceram. Int.* **44**, S199–S202 (2018).
21. Wang, X., Hu, G., Cheng, L., Yang, C. & Wu, W. Comparative study on aging effect in $BiFeO_3$ thin films substituted at A- and B-sites. *Appl. Phys. Lett.* **99**, 262901 (2011).
22. Yan, J., Hu, G. D. & Jiang, X. M. Effects of aging and fatigue in imprinted $BiFeO_3$ film. *J. Mater. Sci.: Mater. Electron.* **28**, 10400–10405 (2017).
23. Damjanovic, D. Ferroelectric, dielectric and piezoelectric properties of ferroelectric thin films and ceramics. *Rep. Prog. Phys.* **61**, 1267–1324 (1998).
24. Jin, L., Li, F. & Zhang, S. Decoding the fingerprint of ferroelectric loops: Comprehension of the material properties and structures. *J. Am. Ceram. Soc.* **97**, 1–27 (2014).
25. Schenk, T. et al. About the deformation of ferroelectric hystereses. *Appl. Phys. Rev.* **1**, 041103 (2014).
26. Genenko, Y. A., Glaum, J., Hoffmann, M. J. & Albe, K. Mechanisms of aging and fatigue in ferroelectrics. *Mater. Sci. Eng. B* **192**, 52–82 (2015).
27. Catalan, G. & Scott, J. F. Physics and applications of bismuth ferrite. *Adv. Mater.* **21**, 1–23 (2009).
28. Morozov, M. I. et al. In-situ structural investigations of ferroelasticity in soft and hard rhombohedral and tetragonal PZT. *J. Appl. Phys.* **118**, 164104 (2015).
29. Rojac, T. et al. $BiFeO_3$ ceramics: Processing, electrical, and electromechanical properties. *J. Am. Ceram. Soc.* **97**, 1993–2011 (2014).

30. Khanal, G. P. et al. Effect of thermal annealing on crystal structures and electrical properties in $BaTiO_3$ ceramics. *J. Appl. Phys.* **124**, 034102 (2018).
31. Wang, Y. et al. $BiFeO_3$ domain wall energies and structures: A combined experimental and density functional theory +U study. *Phys. Rev. Lett.* **110,** 267601 (2013).
32. Menon, N. K. & Krivanek, O. L. Synthesis of electron energy loss spectra for the quantification of detection limits. *Microsc. and Microanal.* **8**, 203–215 (2002).
